# The Environmental Impacts of the Coronavirus

**DOI:** 10.1007/s10640-020-00426-z

**Published:** 2020-05-07

**Authors:** Dieter Helm

**Affiliations:** New College, Oxford, UK

**Keywords:** Climate change, Coronavirus, Deglobalisation, Economic shock, Greenhouse gas emissions, Lockdown, Pandemic

## Abstract

The Covid-19 coronavirus pandemic has resulted in global lockdowns, sharply curtailing economic activity. It is a unique experiment with substantial impacts that will form the agenda for research. There are five sets of questions: the short-term impacts on emissions, the natural environment and environmental policy, including regulations and COP26; longer-term consequences from the deployment of macroeconomic monetary and fiscal stimuli, and investment in green deals; possible further deglobalisation and its impact on climate change and nature; intergenerational environmental impacts including debt and pollution burdens on future generations; and possible behavioural changes to the environment, both positive and negative.

## Introduction

The Covid-19 coronavirus first struck in Wuhan, the capital of China’s Hubei province, at the end of 2019. It quickly spread beyond Asia. The policy responses have mostly been lockdowns of varying intensities and durations. Though there have been many national and even international curfews in the past, perhaps most prominently during the Second World War, there are no examples of lockdowns on a global scale deployed to counter the spread of disease. It is wholly novel to force significant proportions of the populations of the major world economies to stay at home, and mostly indoors.

This is a large-scale experiment which is and will be modelled by epidemiologists for decades to come. It is also a giant economic and environmental experiment. The world economies went from one state to another very quickly, so it is plausible to argue that everything else remained constant. There has been a very significant reduction in economic activity, very large falls in transport by air, road and railways, and with these have come large falls in air pollution and greenhouse gas emissions, as well as reduced pressure on nature. Since the main effects in the first five months of the pandemic have primarily fallen in the northern hemisphere, this coincides with the breeding season for birds and many mammals and reptiles, and for plants too. There is therefore an opportunity to observe what happens when such an abrupt change happens on a global and local scale, and major parts of the economy cease production.

There are at least five sets of research questions. First, what are the short-term impacts and are any of them likely to be enduring? In particular, how great are reductions in global and local GDP and in air pollution? What is the largely anecdotal evidence on the impact of less travel on the wider natural environment, ranging from reduced tourism to less disturbance of wildlife? What do these short-term reductions tell us about the relationship between GDP and emissions?

Second, what are the longer-term economic impacts and, in particular, the environmental consequences of the economic shocks and the policy responses to them and the risk of a longer-term recession or even depression? To what extent will monetary and fiscal policy stimuli be increased? Will these include further support for “green deals” and associated spending on greening transport, energy and agriculture? Will the legacy of the coronavirus be a strong or weaker COP26 agreement, higher or lower carbon prices, a carbon border adjustment, or a greater emphasis on a lower cost of energy?

Third, what will be the impacts on globalisation, which has been a defining feature of the world economy for the last thirty years? Will concerns about extended supply chains lead to a deceleration of globalisation, or perhaps a decline? How will the effects on globalisation play out in the context of the overall declines in GDP and the policy responses? Would a further decline have positive benefits for the environment? Will the coronavirus encourage reshoring of specific industries, notably agriculture, and a greater emphasis on domestic production of food at the expense of a more environmentally benign use of land?

Fourth, what will be the impacts on future generations and how we account for these on the assets they inherit relative to those the current generation is depleting? To what extent will the economic costs of the coronavirus pandemic fall on the next generation and how might these costs impact on the environment?

Finally, will there be behavioural changes in attitudes towards the environment? Will the experience of the lockdowns encourage a shift towards a higher valuation of environmental goods and services relative to other components of household budgets, or a retreat towards narrower, short-term economic considerations as a result of the impacts of debt, unemployment and reduced economic prospects, reflecting an old idea that the environment is a luxury good?

This paper takes each of these sets of questions in turn. Given how early on in the pandemic this is being written, the evidence continues to emerge and will not be comprehensive for some time to come, and especially for the impacts on nature and wildlife. At this stage, it cannot provide answers, but it can set out the issues behind each of the five sets of questions, and these comprise a comprehensive research agenda for environmental economics as it builds on this unprecedented and unwelcome experiment.

## The Short-Term Impacts

The short-term impacts of the policies put in place to limit the spread of the virus on affected economies have been severe. The imposition of lockdowns stopped many activities in their tracks, with particularly large impacts on travel and tourism, the hospitality industry, retail, and the service sector as a whole. Though some sectors have done well as people stockpiled certain goods and turned to online deliveries, the balance has been heavily negative. It is too early to tell what even the initial GDP impacts have been, as statistical evidence lags, but a range of early estimates from the IMF and others point to an unprecedented large and sudden decline, which will last at least as long as the lockdowns, and possibly well into 2021 (International Monetary Fund 2020).

The environmental impacts in the short term are even harder to calculate, though some, like the fall in greenhouse gas emissions and the improvements in air quality, are more instantaneously measurable. The impacts of less effective wildlife and environmental regulation and enforcement, and the delays to many current policy developments, will not be known for quite some time, and in some cases not for several years. These range from poaching to the COP26 outcomes.

Nevertheless, recent advances in satellite and ground-based mapping technologies enable the real-time monitoring of a number of pollution types, notably emissions of greenhouse gases, and urban air quality. Early indications are that there has been a dramatic fall in pollution. Coal-fired power station utilisation, already in decline in most major economies outside China, Japan and India, has fallen back sharply, and especially in China in the early months of the pandemic.^1^ There has been a sudden and sharp decline of transport, and with it the burning of oil. These energy-related emissions reductions are not, however, replicated in agricultural emissions, which do not appear to be significantly affected so far.

It is too early to establish exactly where and by how much the emissions reductions have taken place. Nevertheless, there are early indications that nitrogen dioxide (NO_2_) emissions have fallen by almost half in a number of European cities (European Environment Agency 2020), overwhelmingly the result of the collapse of transport demand. Greenhouse gas emissions fell sharply in February in China, but with the beginning of a rebound from late March. The scale of the falls may have been around 20%.^2^

The most striking correlation in the pandemic is between significant falls in emissions and falls in aggregate demand and aggregate consumption. Contrary to the claim that GDP and emissions have been decoupled (European Commission 2019 and IEA 2016), they have been highly correlated in the coronavirus pandemic in all the major countries affected. China may record a 10% reduction in GDP in the first months of 2020, against a projected 6% increase (International Monetary Fund 2020). Major EU economies may see similar or larger falls. The evidence from the pandemic is that it is not the case that decoupling has occurred at the global level, or even at the European level. Emissions and GDP have both fallen sharply.

The relationship between emissions and GDP is less important than that between the increased concentration of carbon in the atmosphere and GDP, and these concentration numbers are not yet available for the first months of 2020. While emissions are more easily measured from power stations, large industrial plant and transport, the concentration of carbon in the atmosphere is the consequence of the *net* of all the various emissions (including forest burning, and peat and soil degradation) *and* sequestration by natural capital.

When considered in this more relevant context of carbon concentrations in the atmosphere, the impact on emissions during this pandemic may follow the pattern of the last thirty years since the baseline for the emissions reductions targets. Since 1990, the concentration of carbon in the atmosphere has continued to rise at around 2 ppm year on year, and behind this lies the fact that the last thirty years have been the golden age for fossil fuels (Helm 2020). While it is true that measured territorial carbon emissions in the EU have become decoupled from EU GDP, it is also true that the composition of EU production and consumption has changed significantly, and in particular that EU carbon-intensive production declines have been partially offset by EU carbon consumption embedded in imports.^3^ It points towards the uncomfortable conclusion that climate change is unlikely to be mitigated solely by the territorial supply side. Note too that the EU has a static population, whereas global population is rising. It is going to be much tougher if world economic growth resumes to trend and the global population continues to rise. Some aspects of net zero carbon production territorial targets in the UK and EU may even be counterproductive.^4^ The emissions reductions during the pandemic, in reducing GDP sharply, have demonstrated that emissions and GDP are correlated not just globally but even in Europe.

Put simply, the evidence from the correlation between the falls in emissions and GDP during the pandemic lockdowns indicates that achieving the Paris Agreement of a 1.5 °C limit to global warming is going to be very difficult if GDP and population continue to rise. This observation should further ignite the debate about whether growth in consumption is compatible with limiting global warming and protecting the environment more generally, and whether technological progress can be fast enough to reduce the environmental impacts of that greater consumption.^5^ The coronavirus reminds us that consumption is still a critical variable in environmental outcomes.

For the second area of immediate impacts, wildlife and environmental protection, the evidence is largely anecdotal so far, and based on the expected consequences rather than new data. The decline of road traffic will reduce roadkill and allow for more connectivity between animal populations. But it will also reduce environmental monitoring and the enforcement of regulation, even where these are not relaxed. Poaching, wildlife crime and pollution incidents are likely to be less easily detected. Human protection of specific species has declined as the northern hemisphere breeding season for birds and mammals gets under way.^6^ The sharp decline of tourism will reduce footfall in sensitive environmental areas, and may improve breeding success. The reduction of roadside verge-cutting will assist some plants, including native wildflower species. Natural resources, notably in rainforests, will face temporary reductions in demand, but are areas less likely to be affected by lockdowns, not least because enforcement is very difficult. Short-term reductions in ecotourism will have mixed impacts, reducing disturbance, but also reducing the incomes which some countries rely upon for protection, such as Costa Rica’s rainforests and Botswana’s Okavango Delta. On balance, it is too early to tell what the net effects will be, and there is likely to be considerable variance between species, habitats and countries.

For the third area of short-term impacts, policy developments, the most immediate effects of the emissions reductions resulting from the lockdowns are likely to be felt at the now postponed COP26, and on climate change policies in the first half of this decade. Since emissions have fallen in the short term, and emissions in 2020 are likely to be significantly lower than forecast, there will be a temptation to argue that the need for urgent action is correspondingly less pressing, and that in the short term other considerations such as income support and welfare payments should take priority over climate change, and investment should be directed towards health services.^7^ Yet, except in the very short run, it is only an assumption that targets will in fact be easier to meet, especially when the effects of the coronavirus pandemic resulting in a further lowering of fossil fuel prices are taken into account.

In the very short term, the coronavirus has dominated almost all political and administrative bandwidth, with little time to pursue other environmental priorities. In the EU and the UK, the pressing reforms of agricultural and pesticide use are being delayed.^8^ Since they too contain major agricultural and environmental dimensions, delays to the trade agreements with the EU and the US, and between the UK and the EU and the UK and the US are also inevitable consequences of the pandemic. This matters, not least because among these negotiations, Brexit has particularly significant environmental implications, which are yet to be addressed and resolved.

The immediate crisis has been used to relax some environmental regulations, and there has been a weakening of enforcement, not least because of the lockdowns and social-distancing requirements. In the US, fuel efficiency standards have been lowered and environmental enforcement restricted (Utility Dive 2020). Furthermore, when combined with falling oil and gas prices, the lower standards create the conditions for a strong rebound in transport demand and transport-related pollution as soon as lockdown restrictions ease.^9^ If the rationale is cost relief to drivers now, it will have consequences for wellbeing later, for both air quality and for climate change.

## The Longer-Term Economic Impacts and Some of their Possible Environmental Consequences

If the short-term impacts of the coronavirus have revealed the correlation between emissions and GDP, the next question is whether the pandemic will permanently lower the level and growth of GDP, and therefore whether the forecasts for emissions should be lowered, and in turn fed through to carbon plans and budgets to meet targets and related policies in countries badly affected by the virus or by the impacts of reduced global economic activity. Will it be even easier (in addition to the short-term falls in emissions) to achieve the carbon budgets and 2030 carbon targets if there is lower GDP growth, and hence can government ease off on climate change policies in the medium term, and so limit increases in prices for energy, transport and food?

In order to assess the longer-term pandemic impacts on GDP and hence emissions, it is first necessary to work out the counterfactual: what would have happened to GDP levels and growth had the pandemic not happened? Though it will be tempting to conclude that weaker GDP levels and subsequent growth are *caused* by the pandemic, many of the ingredients for financial market falls and recession were already in place by January 2020. It is plausible to argue, as the IMF does (International Monetary Fund 2020), that a significant financial crash was already in the making in the US, China and the EU, that asset values were generally significantly overvalued, that a recession in several major economies was a distinct possibility, and that the monetary policy responses in the summer of 2019 were designed to head this off. Indeed, by January 2020 the EU was already flirting with recession, China’s growth rate was falling back, and forecasts for global economic growth had been lowered. In other words, the coronavirus might have accelerated what would in any event have been a serious economic downturn. Whether this would in fact have happened we cannot know.

One further twist to the counterfactual, with particular environmental consequences, is to recall that oil prices had fallen before the coronavirus had its main impacts in Europe and the US, and before the dramatic falls in oil demand. The decision by Saudi Arabia to increase production, following the failure of the talks with Russia, pre-dates the recognition of the economic seriousness of the pandemic. Low oil prices would probably have materialised in 2020 anyway, and there are good reasons for projecting that oil prices will continue to fall through the coming decade and beyond as the supply side is boosted by increasing global shale production, and the demand side weakened by decarbonisation and the coming of electric cars (Helm 2017a). Attempts by OPEC and OPEC +, such as the agreement in mid-April 2020 to cut output, are unlikely to bring supply down to a level close enough to demand to drive prices back up to 2019 levels.

There will be a lagged demand response to lower oil and gas prices, and expectations of the future profile of oil prices will inform capital goods investments. Once car production and new car demand ramps up again, the trend in 2019 towards sports utility vehicles (SUVs) rather than electric cars may be reinforced by the lower fuel running costs—in the absence of a compensating carbon tax.

Notwithstanding the importance of the counterfactual, and hence the necessary caution about just how big the economic shocks have been relative to what might have happened anyway, there are three main (overlapping) policy options for all the main economies in response to the sharp declines in GDP, each with environmental consequences:

(i) further monetary easing, including lower interest rates and more quantitative easing (QE);
(ii) further fiscal stimuli, with larger government borrowing;
(iii) an infrastructure investment package, concentrated on “green deals”.

### Monetary Easing

Monetary easing started in 2000 as a response to the dotcom crash. Instead of allowing economies to go into recession following the long boom of the 1990s, central banks around the world opted to reduce interest rates towards the zero boundary. Thus began twenty years of economic exceptionalism, with close to zero nominal and negative real interest rates. This in turn created a further asset bubble, concentrated in housing, and another financial market crash followed in 2007/08. In response, monetary policy was further eased with QE, expanding the balance sheets of central banks. Negative real interest rates and QE again inflated asset prices and created a further asset bubble which finally burst in 2020 during the pandemic.^10^

The broader macroeconomic impact of monetary easing has been widely analysed and widely disputed.^11^ Its environmental impacts have been less researched. Monetary easing, as the central banks buy up both government and company bonds, increases asset values, including land, and changes the relationship between present and future values by lowering the time discount rate, reflected in the lower longer-term futures prices for government bonds. Higher land prices change the economics of farming, and with it the impact on the environment of agriculture. The higher the land price, the greater the attraction of marginal lands which are then brought into agricultural production rather than left to nature. It could be argued that they have, for example, exacerbated the negative environmental impacts of the CAP.

The low or negative real costs of debt reduce the incentive to save and increase the incentive to consume. Higher consumption causes higher emissions and greater environmental damage.^12^

The relevant counterfactual is: what would have happened had real interest rates followed the historical norm, and approximated the longer-term growth rate (Borio et al. 2017)?^13^ Suppose the real interest rate had been around 2% between 2000 and 2020. The level of debt and the levels of consumption and savings would have been different, and asset prices would have been significantly lower.

Suppose now that the monetary exceptionalism is continued, or even exacerbated, as a result of the pandemic. All of the above can be expected to be repeated. Asset prices will remain inflated, debt levels will increase, and consumption will dominate savings.

Lower interest rates and QE will both reduce the cost of capital for investments and reduce savings. The former will lower the cost of investing in renewables and nuclear electricity generation, most of which are capital-intensive (typically with zero marginal costs). This cost of capital effect will not, however, offset the fall in oil prices since it applies to *all* technologies and not just low-carbon ones. The outcome will probably depend on what governments do, and whether the lower cost of government borrowing is translated by governments into renewables and nuclear investments, and whether the lower price of oil is offset. In other words, what matters is whether environmental policies are designed to benefit from the monetary conditions central banks create, and by carbon taxes.

### Fiscal Stimuli (and Debt)

All major economies responded to the 2007/08 crisis with fiscal stimuli. Debt levels as a ratio to GDP subsequently rose in China, the US and EU member states. With the exception of Greece and, to a lesser extent, Italy and Spain, none led to a reluctance to lend to governments, and eventually all EU member states saw interest rates fall back to their very low historical levels, supported by QE from the European Central Bank, which vowed to “do whatever it takes” to reduce interest rate spreads (Pisani-Ferry 2014).

Subsequently, the EU countries have tried to limit, and in the case of Germany, eliminate the deficits that arose. But as the world economic outlook darkened in the second half of 2019, many countries abandoned attempts to reduce their deficits. The political cover for greater fiscal expansion was typically cited as investment (though not in the US, where the mechanism was tax cuts). It was argued that investment, backed by borrowing, did not worsen the underlying fiscal position. In other words, a policy U-turn was widely deployed, and now it was argued in the EU and the UK that the aim of balancing the overall budget had been economically inefficient, and greater attention should have been paid to the balance sheet, with investment, asset creation and liabilities fully taken into account. (The US had never seriously attempted to bring its budget back into balance.)

In response to the coronavirus, fiscal stimuli have already been significantly increased in most EU countries, including even Germany, and measures have been taken in the US and China too. The EU has proposed a €500 billion package (around $545 billion), while the US Federal Reserve has announced a $2.3 trillion package. Some of this spending would have happened anyway, as the automatic stabilisers kick in to pay for higher unemployment costs and lower tax revenues. These measures are about increasing aggregate demand and have an underlying Keynesian rationale.

From the environmental perspective, the questions are about the impacts of the spending on GDP, on the split in the impacts between consumption and investment, on how the investment component is spent, and in particular on the willingness of governments to engage with climate change and other natural capital enhancement projects. It is not so much the fiscal stimulus per se that matters for the environment (although it does affect consumption, as discussed above), but rather its composition, and how this reflects on the balance sheet.

Fiscal stimuli will increase the demand for energy, transport and agricultural products. They will also increase the demand for timber, and the derived demand for rainforest products. The demand for products like beef (from the Amazon), hardwood (from all rainforests), hydroelectricity (dams on all the major rivers) and palm oil (from Malaysia and Indonesia) will rise as a result. In other words, the demand for primary natural resources goes up, and the level, and especially the composition, of GDP in the years following the coronavirus will depend on the extent and nature of the fiscal stimuli.

### Green Deals

Mindful of the composition effect of monetary and fiscal stimuli discussed above, some have argued that in a classic demand and supply shock, the obvious policy to pursue is a public infrastructure investment strategy, and that a key part of this should be a new climate change package—a green deal. Some have even referred to this as a new green “Marshall Plan” (European Commission 2019, 2020a, b).

There are two parts to these proposals: claims about the superior economic returns to such investments, considered broadly to also include environmental resilience and distributional impacts, compared with alternative investments; and proposals for funding and financing this expenditure. It is sometimes argued that renewable energy investments, for example, are already cost-competitive with fossil fuel alternatives, or that they will be in the near future. This is a beguiling argument, and a dangerous one. If it is true, then this investment will happen anyway and there is no need for additional subsidies, and hence there is no need for a green deal. Few of the advocates of green deals are willing to accept this conclusion. Alternatively, if it is not true, the justification for a green deal rests in principle on the difference between the higher costs of green investments and the implied carbon price that would have achieved the climate change objectives, notably net zero. The obvious economic policy to close this gap would be to impose the carbon price, domestically and at the border, and then the macroeconomic green deal would again be unnecessary. What is missing is an argument for using government subsidies as opposed to correcting market prices for pollution costs.

If the renewables were not on a path to very quick cost-competitiveness in 2019, the sharp falls of oil, gas and coal prices referred to above have changed the arithmetic further. Worse from the renewables and nuclear perspectives, the costs of fossil fuels, from extraction and refining to transportation, are likely to fall as a result of the price falls as they cascade through the supply chain, through to steel, labour and other suppliers: costs and prices tend to be correlated.^14^ Renewable generation lobbyists are keen to point to the falling costs of renewables, but less willing to carry the assumption over to fossil fuels, where technological progress in the last decade has been extraordinarily fast. Renewables lobbyists also tend to avoid comparing apples with apples, neglecting the system costs of intermittency and decentralised and disaggregated generation (Helm 2017b, chapter 7).

The second part of the argument relates to the relative economic returns as between different types of investment, assuming that an investment stimulus is the correct response to the macroeconomic shocks. The assumed priority for green investments is far from obvious. The returns to road building, for example, can be high, and also to house building, and airports, even if a carbon price is factored in (Highways England 2019; Highways England 2015). Even with a high carbon tax, the costs of fossil fuel-powered cars may be lower than electric ones. The returns to health expenditure have been revealed by the pandemic as much higher than previously anticipated.^15^

In addition to health, the one aspect of the infrastructure whose economics has been markedly improved by the pandemic is communications and, in particular, fibre optics. The switch to video and other forms of virtual communications and work practice during the lockdowns is highlighting the system benefits of full fibre, and points towards an emerging Universal Service Obligation (USO), notably in the UK and rural US.

As long as the total investment budget for government is limited, there will be choices and trade-offs to be made. The green deal investments might not turn out to be the highest priority, though fibre will be important in facilitating the operation of decarbonised electricity and transport systems, and especially the intermittency and the small disaggregated nature of decentralised renewables generation (Helm 2017a, chapters 10 and 11).

The final consideration is where the savings are going to come from to fund the investments, since the monetary stimuli are likely to discourage current savings. Green deal advocates variously argue that this should be borrowing from the future, and hence future savings, or that QE should be utilised to monetarise the costs of the investments. Some envisage a large-scale QE programme, and then link this to a Keynesian argument by claiming that the resulting increase in aggregate demand will multiply through the economy, and therefore pay for itself. This last argument runs into not only the general objections to QE, but also the prioritising of consumption over investment deficit spending to increase demand quickly, and the impact of the higher aggregate demand and consumption on the environment through higher carbon emissions and greater pressures on the natural environment. Linking green investment with Keynesian demand management is perhaps one of the most questionable aspects of the green deal cases. The case for green investment should be considered on a stand-alone basis, not as a stimulus to aggregate demand in the context of a recession. (This has considerable intergenerational impacts, to which we return in Sect. 5 below.)

Whatever the economic arguments for green investments as part of a large-scale public investment programme, in the post-lockdown world there will be other pressing claims on national budgets. In addition to the increased current expenditure to bail out the casualties, public investments will almost certainly be directed to healthcare and, in reflecting the higher death rates among the elderly, to the social care infrastructures. These, plus fibre networks, are likely to be not only economically more attractive, but also, given the nature of electorates and voting behaviours, to have political priority too. Green investments are not the sole component of a sustainable long-term growth strategy.

## Deglobalisation, Trade and Environmental Impacts

One major source of environmental damage to the climate and biodiversity over the last thirty years has been directly and indirectly caused by the rise of China and its phenomenal economic growth, measured in conventional GDP terms. China now burns half the world’s coal (IEA 2019), it has dammed the upper Mekong to provide hydroelectricity, and all three of its major rivers have suffered gross pollution. China’s demand for food and other natural resources has encouraged it to engage in significant investments in Africa and to sponsor large-scale infrastructure along its Belt and Road Initiative, building more dams and more coal-fired power stations, and opening up natural areas.^16^ It is therefore especially important to focus on the impacts of the coronavirus on China and its policy responses.

Much of this pollution has been associated with producing goods for consumption by other countries, and notably by the US and the EU countries, comprising a significant share of world GDP. The Chinese growth model has been much debated, but at its early core has been the export of carbon and energy-intensive goods (Pan et al. 2009). The corollary of this has been the relative decline of home production in the US and especially in the EU of steel, fertilisers, petrochemicals, aluminium and even cement (the big five traded carbon-intensive goods), partly supplanted by the Chinese exports. In other words, much of this pollution in China has been for the benefit of US and EU consumers. Utilising territorial carbon production measures for the climate change targets has disguised this causal relationship, and painted an unduly rosy picture of EU efforts to reduce emissions, while at the same time increasing global warming by increasing the carbon emissions in China and hence the relentless growth in the concentration of carbon in the atmosphere.^17^

One key reason why China has in the past succeeded in gaining export markets is the supply of large quantities of cheap and compliant labour migrating from the countryside to the cities, and being able to extract a considerable savings surplus from them, which the Chinese state has then recycled to investment. Cheap labour has encouraged US and European firms to outsource production to China and then import the products back into their home markets. This has been the case across a wide range of goods, from mobile phones to clothing and footwear. The result has been a globalisation of production and the extension of supply chains. The coronavirus has highlighted the fragility of some of these, and the extent to which the US and the EU are dependent on everything from face masks and medical equipment through to communications technologies.^18^

Some commentators argue that the coronavirus will encourage a retreat to a greater emphasis on national production, and domestic security of supply, which will in turn reduce the pollution from shipping and aviation, and reduce global pollution since environmental standards are higher in the US and Europe generally, and coal is a much smaller proportion of energy inputs to this production. It assumes that globalisation has been bad for the environment, that deglobalisation will improve the environmental outcomes compared with what they would have been, and that the pandemic has caused this deglobalisation. Again, the counterfactual matters in working out what the contribution of the virus will be: the growth of world trade was already slowing in 2019,^19^ and the coming of digital technologies would probably have slowed it even further. Robots replace cheap labour, they do not sleep or require welfare payments, and they do not catch the coronavirus. Economic growth may decouple from the model which relies on locating production close to cheap input costs as opposed to close to customers, with robots and 3D printing playing enabling roles.^20^

The arguments need to be disaggregated. First, there is the general question of the link between globalisation in general and environmental outcomes. Second, there is the question of whether the experience of the virus is causing more deglobalisation. Third, there is the specific question about whether supply security with greater domestic production is always a good thing for the environment.

On the general relationship, the two environment-specific features are: the environmental pollution associated with shipping and aviation (including supporting infrastructures, port facilities and on-land transportation, the extra passenger travel to manage the global supply chains, and the increased tourism that has resulted from China’s globalised growth); and the difference in the composition of factor inputs (especially coal and fertilisers) in production in China versus in the importing country.

The virus has, as noted, reinforced the perceived importance and power of nation states over global institutions. The World Trade Organization appeals body is already not functioning because the US has not nominated a new member to make it quorate, and the widespread adoption of state aids has exacerbated protectionist moves already in place. These impacts should be sufficient to limit the bounce-back of trade and therefore of the associated shipping and aviation demand post-lockdowns. The net environmental impact will also have to take into account the other environmental effects of production in different locations.

One additional positive environmental consequence of the more national approach to trade is that it may encourage a move towards the inclusion of environmental costs, and in particular a border carbon adjustment. The European Commission had already proposed such a measure prior to the pandemic (European Commission 2019). Not to include carbon and other environmental costs in trade is to distort trade, and nowhere is this more apparent than for carbon, where higher carbon prices in Europe have given an additional distorting competitive advantage to imports from China (Helm et al. 2012). It may be that the US will consider returning to a global carbon framework if China is placed on an equal footing. This was the requirement for Obama, and the unequal nature of the commitments has been a major factor in the US Senate’s unwillingness to ratify the Kyoto Protocol and its implicit veto over signing subsequent global climate treaties.

The opportunity presented by the pandemic to argue against trade and for greater national security of supplies has not been lost on lobbyists, and in particular farming groups. The coronavirus arose in the context of the trade war between the US and China, and trade negotiation between the EU and the US, the UK and the EU, and UK and the US. Resilience of supply chains is, however, not the same thing as food self-sufficiency, and the lockdown experience in the UK has been much more concentrated on food supply and logistics rather than food production. The case for a carbon border tax is that it removes distortion to trade; the case for enhanced agricultural production subsidies is about rent capture and the protection of vested interests. The former improves environmental outcomes; the latter typically does not.

## Environmental Dimensions of Intergenerational Equity, the Balance Sheet Approach, and Natural Capital

Climate change and biodiversity loss are essentially intergenerational problems. The impact of climate change, although already manifest in the 1 °C warming, will overwhelmingly fall on future generations. (At 1° warming, the current generation might in aggregate be better off, given the concentration of wealth in the temperate zones and the impact of the warming so far on heating requirements, winter deaths and lengthened growing seasons in agriculture and production generally (Helm 2015a, chapter 1).) The depletion of biodiversity has yet to cause significant global economic losses. Subsequent generations are likely to inherit a climate with over 2° warming and an environment denuded of much of the great biodiversity reservoirs.

In addition to facing the consequences of the current generation’s pollution, future generations are also likely to be endowed with significant debt, and in three parts: the general debt to support consumption levels now in excess of national incomes; the debt associated with new fiscal stimuli to address the negative economic consequences of the current pandemic; and the specific debt to fund investments designed to reduce future pollution.

The coronavirus almost certainly makes intergenerational imbalances worse. In the short term, the young are likely to be disproportionately disadvantaged by the coronavirus lockdown since they are more represented in the leisure, entertainment, restaurant and travel industries (Joyce and Xiaowei 2020). The young largely escape serious health impacts from the virus itself, whereas the old (especially the over-65s) comprise the majority of the deaths. There will be longer-term consequences: the young will also inherit the debts, and the more expansive monetary policy responses may further inflate the prices of key assets such as housing.

The combination of these general impacts of pollution and debt, the additional debt and asset inflation impacts on the next generation arising from the response to the virus itself, and any slackening in political willingness to address the damage to the climate and biodiversity, will together probably cancel out the gains that would be made by applying a lower discount rate to investments which the monetary stimuli will cause. A lower discount rate makes the future more important, and tilts investments towards the longer term, but in practice there is going to be a budget constraint rationing public expenditure, exhausted by health and social care spending and the other consumption-supporting measures to address the immediate crisis. The beneficiaries of higher current expenditures and the health investments are probably going to be more heavily among the current generation—for pensions and end-of-life health costs, for example.

The scale of the intergenerational inequity would be reflected in a national balance sheet which fully incorporated natural capital. With the pollution costs assigned to those who cause the pollution (the polluter-pays principle), and the capital maintenance to preserve natural capital intact charged to the current budget line, and enhancements added to the assets against the liability of debt for these enhancements (and not for current spending), the scale of the inequity would be made apparent.^21^ The absence of full balance sheet national accounts obscures the scale of the inequity.

While there is a general shift towards balance sheet accounting, with assets and liabilities and hence better representation of the state of the economy and the intergenerational elements, there is no reason to assume that the experience of the coronavirus will encourage this approach and its policy implications to be implemented. On the contrary, the scale of government spending and borrowing will more likely lead to a host of accounting tricks to disguise the numbers.^22^ Even here there will be limits, since shifting the costs off the balance sheet does not make them go away. The conventional route has been to privatise, applying the costs as user charges. It is, however, questionable as to whether, post lockdown, there will be a political appetite to allow electricity, water, transport and even communications bills to rise so as to incorporate these costs.

## The Permanent Impacts: Behavioural Shifts

There have been claims that the experience of the coronavirus will change behaviour and personal and political choices. Some think this will lead to more aggressive action at COP26 and a greater willingness to tackle biodiversity loss. Others take the opposite view, arguing that the reduced incomes will shift priorities to the short term and measures to boost consumption. To understand how the experience of the virus might change behaviour, the economic theory of choice is the obvious starting point.

One of the longest-running disputes in economic theory is about the assumption of exogenous preferences and their translation through the axioms of choice to a rational preference ordering. The *exogenous* preferences, plus the *exogenous* technologies, set the framework for the neoclassical theory of demand and supply and for the derivation of the general equilibrium.^23^ The reason the assumption of exogenous preferences is so important is that it draws a line between psychology and economics. Once accepted, choice can change only with changes in information. It is pointless to try to change the underlying psychology since it is assumed away.

The relevance of all this for environmental policy and the impact of the coronavirus is that there has undoubtedly been a major change in information. The shock of the virus itself and the daily news and social media (Shiller 2019) has focused attention, and the experience of lockdowns has added information. The old adage of “you don’t know what you’ve got till it’s gone” applies: now large numbers of people do know what the absence of nature and the natural world means as they are confined to their homes. They have also experienced a sharp decline in road traffic and aviation, and many have experienced much cleaner air for the first time. These various informational impacts and novel experiences may have longer-lasting effects.

This is not the only experience that the virus has brought. There has been widespread anxiety and considerable insecurity as conventional assumptions about the security of jobs and the value of savings and pensions has been challenged (Office for National Statistics 2020). Some food items have been temporarily scarce and there have been interruptions to the food supply chains. Some people have even gone hungry. The experience of negative shocks may make people more risk-averse, saving more, while lower actual and expected income may reduce the willingness to pay for environmental improvements. The tax base has been eroded, making the public goods particularly vulnerable, and increases in taxation are widely anticipated.^24^

The empirical issue is the extent to which income (and subjective insecurity) and the willingness to pay for environmental goods and services are correlated. In the context of the coronavirus, will lower incomes lead to a lessening of the demand for better environmental outcomes, or will the experience of losing access to nature make it more highly valued? More specifically, are there environmental assets, like town parks and urban green spaces, that are now likely to be more highly valued.^25^

So much for information. What about preferences? There have been major challenges to the exogenous preference assumption, with psychologists pointing to the scope for changing endogenous preferences. For given information, will the experience of the virus make people prefer higher or lower environmental outcomes over time, independent of the new information. Will they have acquired a stronger preference for nature? In particular, because the virus is global, will the experience make people more minded to support global action on other problems, notably climate change and in respect of COP26, and more community-spirited on a local basis? Or will the reactions to the virus encourage more nationalism, and less globalism and less community as people learn to distance themselves from social interactions?

There are at least two problems with endogenous preferences. The first is that the malleability of human nature is at best a long-term project. Human nature tends to be fixed, and this is a good working assumption, especially for policy design and implementation. Second, it is not clear why changes in human nature should lead to stronger preferences for better environmental outcomes. Might not the opposite be the case?

Many environmental problems are urgent and cannot wait for education and other public pressures to change people’s preferences. Global warming of 3° or more by 2100 is unlikely to be headed off by changing human nature between now and then, whether or not it is possible or even desirable.

The response to the virus provides a good illustration of the importance of focusing on preferences as they are, rather than as some would like them to be. In due course, it will be possible to work out the cost of saving lives from this coronavirus compared with other causes of premature deaths, like famine, poverty and diseases such as malaria, and especially the cost of preventing wars like those in Syria and Yemen. This partiality of human nature is one important lesson the pandemic has illustrated, and it is the context for effective environmental policies.

These narrower revealed preferences from the virus experience have a particular resonance in concerns about climate change and biodiversity loss. It has been argued, notably by Stern (2007), that the economics of climate change should be assessed on the assumption of zero discounting of future utility. This is a key assumption necessary to reach his conclusions about the economic attraction of taking action now. He relies on a classic utilitarian argument that we should not discriminate between people according to the time they live, quoting Frank Ramsey (1928). It is hardly convincing: there is no evidence whatsoever that human nature leads us to treat those people in Yemen or Syria, or those who starved in Darfur, on a par with citizens of our own countries. Aid budgets tend to be less than 1% GDP. Of course, just because something “is”, does not imply it “ought” to be, but if the “ought” bears no relation to human nature, it is a very poor basis for public policy towards climate change or biodiversity. It would also cut across democracy, since democracy relies on actual preferences, not idealised ones.

## Conclusions

The impacts of the coronavirus will be argued about for decades to come. It may turn out to be one blip in the long battles between humans and viruses throughout human history, just as Spanish Flu was largely forgotten by historians in the 1920s and 1930s. Humans live with viruses and will have to learn to live with this particular coronavirus. Though there have been short-term environmental gains from the reductions in emissions and the consequent improvements in air quality, some of these will probably prove temporary, if and when normality returns and GDP rebounds, aided by major monetary and fiscal stimuli.

While it is important not to overplay its longer-run impacts, the coronavirus provides valuable research evidence into the causes of pollution and, in particular, the impacts of the great experiment of a sudden stop to quite a lot of economic activity, and notably to transport. The most important lesson from the virus so far is that pollution and GDP are still correlated, not decoupled. It is hard to overstate the importance of this lesson to the design of policies aimed at mitigating climate change and biodiversity loss. The scale of technological progress in the next thirty years will have to be extraordinary, and to be accompanied by immediate translation into investments to change the capital stocks, if global GDP growth of even 3% per annum and another billion people are to be accommodated, and global warming is to be limited to 1.5°. That technological progress would also need to try to replace the natural resources being depleted in the major biodiversity hotspots, primarily the great rainforests.

The macroeconomic policies are likely to focus on consumption rather than investment, to perpetuate the mis-valuation of assets through QE and negative real interest rates, and favour spending on health and related public services rather than the environment. These will benefit the current generation—and specifically its older members—disproportionately compared with the interests of the next, widening intergenerational inequity.

Grounds for optimism are harder to find, but one stands out: the reduced enthusiasm for globalisation, and the corresponding falls in carbon-intensive global trade, and the shipping and aviation and associated carbon-intensive infrastructure. To these possible gains might be added the speeding-up of the roll-out of digital technologies which render the attractions of commuting and work-related travel less appealing, making way for a more digitally enabled, decentralised economy.

Although many of the likely impacts of the pandemic may not be environmentally benign, they should not distract from the economic arguments in favour of tackling climate change and biodiversity loss. The economic case for improving air quality, for addressing marine pollution and for closing down coal-burning and peat losses remain, as does the case for the electrification of transport. The facts have been marginally changed by the virus, and while these changes are not sufficient to make us change our minds, they should help to recalibrate the answers, and this is aided by the one great benefit of the coronavirus—an explosion of empirical data from the shocks, on which a new research agenda can and should be grounded.

## Footnotes

1 See the FT coronavirus tracker at https://www.ft.com/content/0c13755a-6867-11ea-800d-da70cff6e4d3

2 https://www.ft.com/content/0c13755a-6867-11ea-800d-da70cff6e4d3.

3 On the difference between carbon production and carbon consumption, while territorial emissions are relatively straightforward (outside those from soils, trees and peat), for consumption the composition of the carbon content of imports is much more complex. Nevertheless, in the UK, for example, it is estimated that carbon consumption is 70% higher than carbon production (Department of Environment, Food and Rural Affairs; Helm 2017b, chapter 2).

4 The Committee on Climate Change (2019) erroneously claimed, for example, that “By reducing emissions produced in the UK to zero, we also end our contribution to rising global temperatures” (p. 8). We will never get to zero emissions (nor should we), and as long as there are imports, net zero does not end the contribution to climate change (Helm 2020).

5 The convenient assumptions behind the Environmental Kuznets Curve, and modern economic growth theory, which posits substitutability between natural, man-made and human capital and labour, are not playing out for climate change or biodiversity, and the alternative approach of placing natural limits and constraints on such models requires extraordinarily rapid gains in technology to offset the potential economic losses as the constraints bite.

6 Examples include the surveillance and protection of nests of critically endangered species primarily in the northern hemisphere. In the UK these include the prevention of predation by foxes and corvids of the little tern colony on Chesil Beach, and the surveillance of key sites of raptors, including hen harriers.

7 For Germany for example, it might cover up the failure to reach its 2020 targets (Helm 2020), while in the UK, where carbon budgeting is most advanced, the existing third, fourth and fifth carbon budgets will be easier to meet.

8 In the UK the Agriculture and the Environment Bills have been delayed, and new statutory environmental targets will not be set for some time to come. The new Office for Environmental Protection is now scheduled for 2021 (Euractiv 2020).

9 The continued emphasis on social distancing may also encourage a switch from public transport to the private space of cars.

10 A particular feature of the post-2007/08 financial markets was the increasing reliance on share buy-backs and further switching of debt for equity (Philippon 2019).

11 See summary and references at Cui and Sterk (2019), and Oxford Review of Economic Policy (2018).

12 On the savings issue, see Dasgupta (2019), Arrow et al. (2012) and Shunsuke and Pushpam (2018).

13 For recent data, see https://data.oecd.org/interest/long-term-interest-rates.htm.

14 The price falls in late 2014 were assumed by the Saudis to render US shale oil uneconomic at about $70/barrel. However, the impact of the price falls was to reduce the costs of production to around $35/barrel (Helm 2017a).

15 Although air quality improvements improve health outcomes and the environment generally, there are lots of health expenditures with stand-alone justifications.

16 Prior to the virus, it had 148GW of new coal under construction at home, and 16GW in Pakistan. See Carbon Brief (2020) and BBC (2019).

17 It has also resulted in significant implicit subsidies to high carbon emitters in the EU, by handing out free EU ETS permits.

18 Experience of supply chain dependency during the pandemic comes in the context of the political interventions in respect of 5G networks made by Huawei.

19 See for background Ortiz-Ospina and Beltekian (2018).

20 See Ford (2015). For an alternative view see World Trade Organization (2018).

21 See Mayer (2013), and Helm (2015b), chapter 3. See also Office for National Statistics UK national balance sheet estimates, available at https://www.ons.gov.uk/economy/nationalaccounts/uksectoraccounts/bulletins/nationalbalancesheet/previousReleases

22 It is likely that more private finance initiatives, public–private partnerships and special purpose vehicles will be utilised to massage the overall scale of the intergenerational deficit.

23 The ordinal theory of choice was set out by Hick (1939). On revealed preference see Sen (1971).

24 This is where the neo-Ricardian effect may come into play (Barro 1974).

25 Since many urban green spaces have greater biodiversity than national parks, these specific asset enhancements might increase overall biodiversity too. On the value of natural capital close to urban populations see Brander and Koetse (2011).
